# Dislocation arrangements in 4H-SiC and their influence on the local crystal lattice properties

**DOI:** 10.1107/S1600576723003291

**Published:** 2023-05-29

**Authors:** Melissa Roder, Johannes Steiner, Peter Wellmann, Merve Kabukcuoglu, Elias Hamann, Simon Haaga, Daniel Hänschke, Andreas Danilewsky

**Affiliations:** a Crystallography, Albert-Ludwigs University Freiburg, Hermann-Herder Strasse 5, Freiburg, Baden-Württemberg 79104, Germany; b Crystal Growth Lab, Material Department 6, University of Erlangen-Nürnberg, Martensstrasse 7, Erlangen, Bayern 91058, Germany; c Institute for Photon Science and Synchrotron Radiation (IPS), Karlsruhe Institute of Technology (KIT), Hermann-von-Helmholtz-Platz 1, Eggenstein-Leopoldshafen, Baden-Württemberg 76344, Germany; HPSTAR and Harbin Institute of Technology, People’s Republic of China

**Keywords:** 4H-SiC, dislocations, synchrotron white-beam X-ray topography, high-resolution X-ray diffractometry, crystal growth, reciprocal-space maps

## Abstract

Two wafers of one 4H-silicon carbide (4H-SiC) bulk crystal, one cut from a longitudinal position close to the crystal’s seed and the other close to the cap, were characterized with synchrotron white-beam X-ray topography (SWXRT) in back-reflection and transmission geometry to investigate the dislocation formation and propagation during growth. For the first time, full wafer mappings were recorded in 00012 back-reflection geometry with a CCD camera system, providing an overview of the dislocation arrangement in terms of dislocation type, density and homogeneous distribution. Furthermore, by having similar resolution to conventional SWXRT photographic film, the method enables identification of individual dislocations, even single threading screw dislocations, which appear as white spots with a diameter in the range of 10 to 30 µm. Both investigated wafers showed a similar dislocation arrangement, suggesting a constant propagation of dislocations during crystal growth. A systematic investigation of crystal lattice strain and tilt at selected wafer areas with different dislocation arrangements was achieved with high-resolution X-ray diffractometry reciprocal-space map (RSM) measurements in the symmetric 0004 reflection. It was shown that the diffracted intensity distribution of the RSM for different dislocation arrangements depends on the locally predominant dislocation type and density. Moreover, the orientation of specific dislocation types along the RSM scanning direction has a strong influence on the local crystal lattice properties.

## Abstract 2

In this work, the determination of dislocation arrangements in 4H-silicon carbide (4H-SiC), their evolution in the growth direction and their influence on the crystal lattice properties are presented.

## 1. Introduction

When considering the physical and chemical properties of silicon carbide (SiC), its importance for high-power devices becomes apparent. In particular, compared with silicon (Si), SiC has a nearly three times higher band gap, a higher breakdown field and a higher thermal conductivity (Bhat, 2010 ▸; Levinshtein *et al.*, 2001 ▸). Moreover, SiC has been regarded as functioning well under high-temperature, high-power and high-radiation conditions at which conventional semiconductors, like Si, cannot perform adequately (Bhat, 2010 ▸). Despite the large number of applications, the variety and density of dislocations in SiC is still high compared with state-of-the-art Si. The main growth challenges include high temperatures above 2000°C and the conservation of large axial temperature gradients without fluctuations (Kimoto, 2016 ▸). A low crystal quality leads to a strong degradation of the device’s performance or even total failure (Neudeck *et al.*, 1997 ▸; Malhan *et al.*, 2003 ▸). Although the growth techniques and crystal quality of 4H-SiC, as one of the most important polytypes, could be improved, various inhomogeneously distributed dislocation types are still present in currently available commercial wafers. To reduce the dislocation density permanently, a fundamental understanding of the properties, interactions and development of the different dislocation types is important. In previous work, basal-plane dislocation (BPD) networks were shown to occur solely in wafer areas with the highest simulated resolved shear stress, and small-angle grain boundaries (SAGBs) were identified to consist of threading edge dislocation (TED) arrays arranged along [1 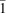 00] in the direction of the wafer’s misorientation (Steiner *et al.*, 2019 ▸). In this work, we focus on the determination of dislocation arrangements, their evolution in the growth direction and their influence on the crystal lattice properties, characterizing two wafers – one from close to the crystal’s seed and the other from close to the cap – of a bulk 4H-SiC crystal. The crystal was grown by physical vapour transport, which is most suitable for the growth of SiC used for applications in power electronics (Wellmann *et al.*, 2015 ▸; Wellmann, 2017 ▸, 2018 ▸).

**Figure 1:**
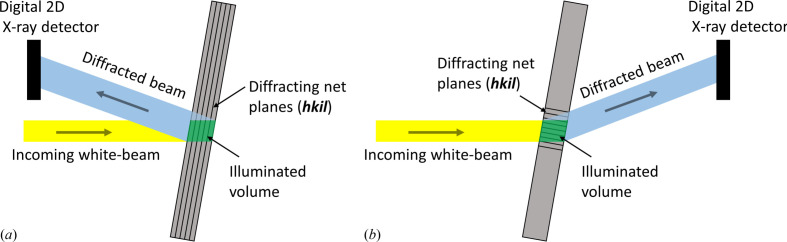
An illustration showing the principle of SWXRT in (*a*) back-reflection and (*b*) transmission geometry.

For material characterization we applied synchrotron white-beam X-ray topography (SWXRT), which is a well suited imaging technique for defect characterization and recognition in large bulk crystals (Tuomi *et al.*, 1974 ▸). The SWXRT characterization of the most relevant dislocation types in SiC has been described by Huang *et al.* (1999 ▸), Dudley & Huang (2000 ▸) and Dudley *et al.* (2009 ▸) in detail. In principle, SWXRT employs a wide, parallel and polychromatic X-ray beam, which impinges on the crystal producing a set of Laue spots, each of which corresponds to a specific atomic net plane (*hkil*) fulfilling the Bragg condition for a suitable wavelength (Fig. 1 ▸). In each spot, a projection image of the illuminated sample volume is formed, with contrast corresponding to local lattice deformations associated with crystalline defects like inclusions, stacking faults and dislocations. On the basis of the two major dislocation contrast mechanisms, namely orientation and extinction contrast, the images of X-ray topographs can be interpreted the following way (Authier, 2008 ▸; Bowen & Tanner, 1998 ▸): higher local intensities within the diffracted-beam profile are usually related to locally defective regions, while a uniform intensity distribution indicates a good crystal quality. Moreover, on the basis of the ‘extinction rule’, different dislocation types can be distinguished. Additionally, a strong orientation contrast points to strong crystal lattice imperfections, which mainly arise from a high mosaicity, sub-grains or domain boundaries (Bowen & Tanner, 1998 ▸). For the present work, the different dislocation types and their densities within the investigated 4H-SiC wafers were characterized with SWXRT in both transmission and back-reflection geometry. In our case, the latter is particularly well suited for the identification and characterization of individual threading dislocations (TDs) in the (0001)-cut wafers because they appear as white circular spots, which can be assigned to their TD type according to their different size, shape and diameter (Huang *et al.*, 1999 ▸; Dudley & Huang, 2000 ▸; Dudley *et al.*, 2003 ▸). In contrast, transmission topographs can reveal or confirm the appearance of dislocation types like BPDs, stacking faults, partial dislocations, inclusions and voids. Generally, in transmission geometry, the complete sample volume is accessible and thus transmission topographs give information about the dislocation content through the whole depth of the wafer, while back-reflection topography usually employs rather large Bragg angles, *i.e.* low X-ray energies, and thus accesses mostly information about the crystal regions close to the wafer’s surface. However, in transmission topography, assigning TDs to their type is hindered because superscrew dislocations like micropipes (MPs) appear as two short and parallel dark contrast lines, which only give information about their position in the measured topography area (Dudley *et al.*, 1995 ▸; Vetter & Dudley, 2006 ▸; Guo *et al.*, 2016 ▸). Thus, for this work, the back-reflection wafer mappings were used to assign TDs to their type, and transmission topographs were consulted to confirm different dislocation types or to compare the depicted dislocation density. Additional complementary information about the influence of different dislocation arrangements on the crystal lattice was investigated by means of high-resolution X-ray diffractometry (HRXRD) measurements. Moreover, reciprocal-space maps (RSMs) were used; these are 2D maps of the reciprocal lattice points that allow one to separate the local crystal lattice strain and tilt for certain dislocation arrangements.

## 2. Stress and tilt calculations

Crystal lattice strain is a unitless relative measure given by a mismatch between lattice planes. It expresses the variation of the crystal structure caused by an external force. To calculate the related stress, the strain value has to be multiplied with Young’s modulus (*E*_*hikl*_) (Nye, 1985 ▸). Mayer *et al.* (2003 ▸) presented a way to calculate a non-orientation-dependent Young modulus for 4H-SiC using the elastic constants *c*_*ij*_. Following this calculation, a Young modulus of 442.1 GPa was estimated for the elastic constants given by Madelung (1982 ▸). Strain from RSMs was converted into stress and further tilt values were calculated. With the aid of the diffraction vector (r*), which corresponds to the reciprocal lattice vector perpendicular to the lattice planes (with spacing *d*_*hkil*_), the lattice parameter can be calculated (Nye, 1985 ▸). If the tip of r* is shifted perpendicularly, the lattice planes are tilted, and if the length of r* changes, the lattice plane spacing changes. In RSMs, r* can be estimated by estimating the position of the ‘intensity emphasis’ of each diffracted intensity distribution maximum in relation to the lower diffracted intensity areas in the *q*_⊥_ and *q*_||_ coordinate system (Tonn, 2008 ▸). The minimum and maximum values of the isolines [*q*_⊥_(max), *q*_⊥_(min), *q*_||_(max), *q*_||_(min)] that enclose the intensity emphasis will be used for the calculation of the experimental diffracted intensity distribution maxima as follows:

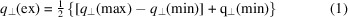

and

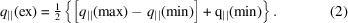

The absolute values for the diffraction vector r*_n_(ex) of each diffracted intensity distribution maximum can be calculated as follows:

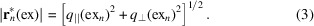

By having the values for the diffraction vector of each diffracted intensity distribution maximum, the crystal lattice strain between them can be calculated (Tonn, 2008 ▸). Furthermore, the tilt angle (ɛ) between two sets of lattice planes can be calculated:

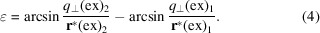

If ɛ > 0°, the diffraction vector and lattice plane are tilted clockwise, and if ɛ < 0°, they are tilted anticlockwise (Nye, 1985 ▸).

## 3. Experimental

The SiC ingot with a diameter of 100 mm that was used for the investigation was grown at temperatures of ∼2000°C in an inductively heated carbon crucible. The growth duration was 79 h, the growth rate was 260 µm h^−1^ and the cooling duration was 40 h. A (0001)-oriented seed with 4° off-orientation was used. The first wafer (A1), cut from close to the crystal seed, and the second wafer (A2), close to the crystal cap, with a distance of 9.4 mm between them, were characterized and compared. The two wafers had a thickness of ∼2 mm and were double side polished. The SWXRT experiments were performed at the topography station of the imaging cluster at the Karlsruhe Institute of Technology (KIT) synchrotron light source (Rack *et al.*, 2009 ▸). Here, wafers with a diameter up to 450 mm can be mapped, taking advantage of the wide X-ray beam generated by a bend magnet source and an optimized X-ray imaging detector system. With an electron energy of 2.5 GeV and a wavelength in the hard X-ray regime above 6 keV, the beamline is well suited for topography. For the measurements in transmission geometry, the 11 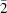 0 reflection was selected at a Bragg angle of 7°, corresponding to 33.82 keV. In order to also enable SWXRT high-resolution full wafer mappings in back-reflection geometry, the topography instrumentation was suitably adapted and extended. For the measurements in back-reflection geometry, the 00012 reflection was selected (Bragg angle around 80°, corresponding to 7.47 keV), minimizing the resulting geometrical image distortion. The wafers were mounted on a stack of three goniometers, which provide tilt and rotation for aligning the sample so that the selected *hkil* reaches the camera system. A sample–detector distance of ∼100 mm was used in order to avoid an overlap of the selected Laue spot with strong adjacent spots, while still preserving sufficiently sharp diffraction image contrast, and a beam size of 5 mm × 7 mm was set.

All topographic images were digitally recorded by using an indirect 2D detector system, consisting of a 200 µm thick LuAg:Ce scintillator crystal coupled by magnifying visible-light optics (Nikkor 180/2.8 ED 3.6× magnification) to a CCD camera (pco.4000, 4008 × 2672 pixels and 9 × 9 µm pixel size), resulting in an effective pixel size of 2.5 µm. The goniometer moves in indicated steps and a reflection pattern of each wafer area is recorded. For each wafer mapping, ∼800 single images were recorded. The collected topographic images show dislocation features with varying degrees of black and white contrast. The topographic images were postprocessed using *ImageJ* (Schneider *et al.*, 2012 ▸) to enhance the contrast, perform a brightness correction, and invert the displayed image grey-value contrast to be consistent with X-ray photographic films where white and black areas indicate not diffracted and diffracted intensities related to locally defective regions, respectively. The X-ray penetration depth for the SWXRT back-reflection topography in 00012 is 4 µm and for the RSM measurements in 0004 it is 0.7 µm. Both values require the assumption of a nearly perfect crystal. For crystals containing defects, the actual penetration depth may be larger. On the basis of the recorded SWXRT back-reflection mappings, the HRXRD measurements were executed on selected wafer areas, representing dislocation arrangements with different characteristics in terms of type, density and homogeneity. The experiments were carried out with the aid of a Seifert XRD 3003 PTS diffractometer, whose optical path consists of a multilayer mirror, a Du-Mond Bartels monochromator and, for the recording of RSMs, an analyser (Jauß *et al.*, 2012 ▸). Its Cu X-ray tube has a long fine focus of 0.4 × 12 mm, which results in a real focal longitudinal area of 5 × 12 mm on the sample. The Du-Mond Bartels monochromator, with two asymmetrical channel-cut Ge crystals adjusted for the 440 reflection, allows only Cu *K*α_1_ radiation to pass and therefore ensures the high angular resolution (DuMond, 1937 ▸; Bartels, 1986 ▸). For the recording of RSMs, the analyser, which consists of a symmetrical (110) channel-cut Ge crystal, was set in front of the detector. On each selected wafer area, an RSM was recorded in the symmetric 0004 reflection. The angular range for ω and 2θ was set to 0.75° with an angular resolution of 0.005° and a counting time of 1 s per step for all measurements. This wide angular range for the recording of RSMs allows one to record signals that occur at a large distance from the main diffracted intensity distribution, generated by dislocations or networks that cause a large stress or tilt, such as, for example, SAGBs. The final image range of all the RSMs is scaled to show the diffracted intensity distribution at the highest possible resolution. Nevertheless, RSMs showing a similar result are scaled equally for a better comparison.

## 4. Results and discussion

### 4.1. Dislocation determination and large-scale distribution

**Figure 2:**
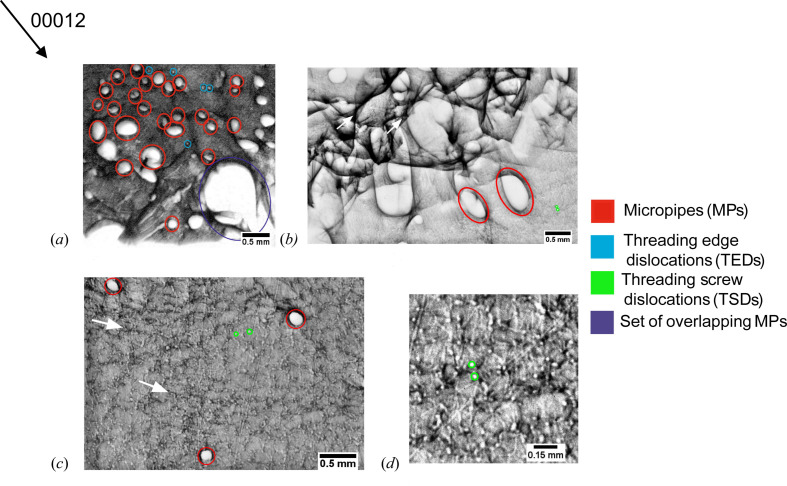
Sections of different 00012 back-reflection topographs of the investigated crystal, showing the different dislocation features in detail. TDs are always depicted as white circular spots with a dark border. (*a*) White spots with the largest diameter and a strong contrast are MPs. In contrast, small-diameter white spots with a weak contrast are TEDs. (*b*) Strong-contrast linear features can be assigned to SAGBs (white arrows). (*c*) Weak-contrast linear features building up a dislocation network are BPDs (white arrows). (*d*) Zoomed-in section showing single screw dislocations, which are depicted as white spots with the smallest diameter and a strong contrast.

**Figure 3:**
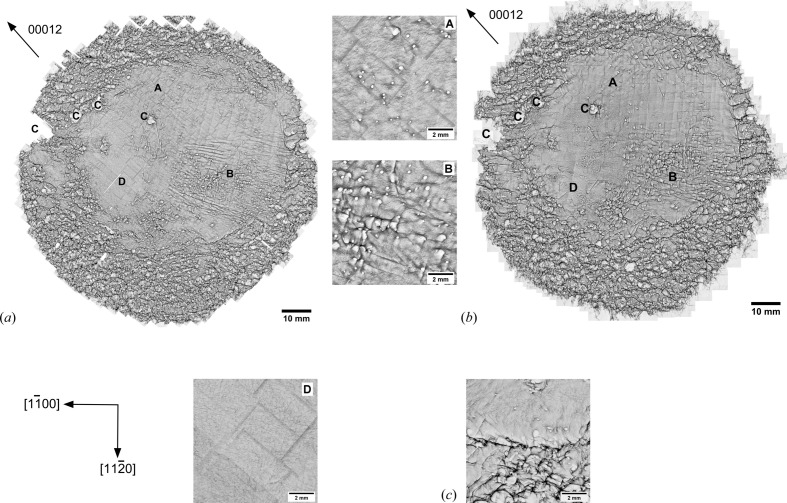
00012 back-reflection topography wafer mappings of wafers (*a*) A1 and (*b*) A2, giving a dislocation overview indicating no change in dislocation arrangement within 9.4 mm of growth. Capital letters A–D mark the position of wafer areas which are made up of specific dislocation arrangements (for a detailed description see the main text). These areas are preserved during growth. In (*c*) the transition from the BPD to the SAGB-dominant area is shown.

As shown in Fig. 2 ▸, in back-reflection topographs all types of TDs are depicted as white circular spots, with a dark border representing their surrounding strain field (Dudley & Huang, 2000 ▸). Depending on the size, contrast and circularity of these spots, they can be assigned to either MPs, threading screw dislocations (TSDs) or TEDs. MPs refer to the white spots with the largest diameter (up to 500 µm) and a strong contrast arising from their large internal strain field [Fig. 2 ▸(*a*)]. TSDs have a comparable contrast but a significantly smaller white-spot diameter (10–30 µm) than MPs [Fig. 2 ▸(*d*)]. TEDs have a comparable white-spot diameter to TSDs but have a weak contrast. Additionally, dislocation networks like BPDs or SAGBs can be identified. BPDs show up as a regular linear network with a comparable weak contrast [Fig. 2 ▸(*c*)] and SAGBs appear as an accumulation of linear features with a strong contrast [Fig. 2 ▸(*c*)]. Pure edge character BPDs with a Burgers vector of **b** = 〈11 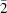 0〉 and a propagation along (0001)-glide planes should appear to be extinguished in the 00012 reflection topographs. Because a BPD network is visible in both 00012 back-reflection wafer mappings, the line vector (**l**) and/or Burgers vector (**b**) presumably consist of an **l** component. However, Huang *et al.* (2007 ▸) reported that a clear contrast of BPDs in topography is also possible for the diffraction vector perpendicular to the Burgers vector. Taking this into account, pure but also non-pure BPD networks should both be depicted in the topographs. The network shown in the wafer mappings is not necessarily a pure BPD network but will be identified as such here, as this is also done in the literature by Dhanaraj *et al.* (2010 ▸). Furthermore, there are white spots visible with a strong non-circular outline and a diameter too large to be counted as one single MP. These features can be assigned to either a group of overlapping white spots forming an MP cluster, a void or a different SiC polytype, which was identified with the aid of crossed polarized microscopy as shown by Presser *et al.* (2008 ▸). Since it is assumed that all types of TDs propagate mainly in a growth direction along the **c** axis, it is expected that wafer A1 will show a similar dislocation arrangement concerning the TDs to wafer A2. The wafer mappings of both, recorded in the 00012 reflection, show a similar dislocation arrangement [Figs. 3 ▸(*a*) and 3 ▸(*b*)]. Both wafers exhibit a wide variety of dislocation types, which are inhomogeneously distributed and show the highest density at the wafer’s border. There are also different wafer areas, which consist of an identical dislocation arrangement in terms of type, density and distribution.

The distinct wafer areas labelled in Fig. 3 ▸ by capital letters ranging from ‘A’ to ‘D’ are preserved along the growth direction from A1 to A2. In regions denoted as ‘A’, the white spots, which represent the appearance of MPs, are arranged at a certain distance from each other. They show a good circularity and an equal border contrast. However, the white spots in regions labelled ‘B’ show a rather elliptical shape and a stronger contrast at one side of their border. The elongation of this enhanced dark border contrast shows a clear preferential direction. These white spots correspond to MPs that appear to have a stronger inclination from the **c** axis, which was confirmed with the aid of crossed polarized *Z* stacks. Regions marked with ‘C’ show an irregularly shaped large-diameter white-spot cluster. Correlating the information from crossed polarized microscopy, it was verified that the white-spot clusters near the wafer middle correspond to MP clusters, whereas those at the wafer’s border were identified as a different SiC polytype, as a residual of the polygonized border. As shown in previous work, a BPD network is visible and limited to the inner ring between the wafer’s centre and its border, which correlates to the highest shear-stress distribution inherent at growth temperatures around 2000°C (Steiner *et al.*, 2019 ▸). Approaching the wafer’s border there is a spontaneous change of the area containing a BPD network to one showing a domination of SAGBs in connection to MPs [Fig. 3 ▸(*c*)], which arise and propagate from the crystal’s seed. This is in good agreement with a strong decrease of basal-plane shear stress obtained from simulations (Steiner *et al.*, 2019 ▸). Thus, BPDs are a good indicator for the inherent shear-stress value. Region ‘D’ on wafer A1 [Fig. 3 ▸(*a*)] shows an isolated BPD network in a circular arrangement, which is clearly separated from the surrounding wafer regions and dislocations. This area can be assigned to the (0001) facet region, which is shifted from the centre due to 4° **c** axis off-orientation. The first change of a wafer area following the growth direction from A1 to A2 is visible on ‘D’, which shows a dissolution of its original circular arrangement and an interruption by MPs that is caused by changing growth conditions. The majority of dislocations at the wafer’s border consists mainly of a high density of SAGBs and MPs [Figs. 3 ▸(*a*) and 3 ▸(*b*)]. The high density of these cause an overlap, and thus the identification of each individual MP is not possible. The SAGB network on wafers A1 and A2 shows a clearly pronounced preferential direction along [1 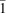 00], which corresponds to the wafer’s off-orientation. In previous work, it was shown that BPDs propagate in the [11 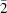 0] direction (Steiner *et al.*, 2019 ▸). Furthermore, both wafer mappings show a strongly pronounced orientation contrast at their border, indicating that the pattern was diffracted outside the wafer border, which points to strong crystal lattice imperfections mainly arising from the presence and high density of SAGBs.

With the aid of full SWXRT wafer mappings in back-reflection geometry, an overview of dislocation types and their distribution can be given. The wafer mappings of both wafers, although 9.4 mm lies between them, show the same overall dislocation arrangement and inhomogeneous distribution. For both wafers of the crystal, the majority of dislocations are located at the wafer’s edge, mostly in the form of SAGBs in connection with MPs. The formation of SAGBs arises presumably from strain at the polycrystalline border (Chen & Dudley, 2007 ▸). A change of growth conditions during growth is indicated by the change in the facet region ‘D’ from A1 to A2. However, the dislocation arrangements within the wafer areas ‘A’ to ‘D’ and the position and outline of the SAGBs stay consistent from A1 to A2. Thus, SAGBs are pinned and not influenced by changing conditions during growth. However, small changes within the dislocation arrangement are visible concerning the shape and position of individual MP’s white spots. One option for the local and abrupt propagation changes of MPs is that they can be caused by a curvy phase boundary. Besides a global strong curvature of the phase boundary, there are also localized areas that show strong inclination towards or against the global curvature. Depressions in the boundary and growth valleys may cause dopant inhomogeneities and/or trap them in their valley (Lu & Bauser, 1985 ▸). These curvatures or depressions can cause the MPs to bend. Another option is the influence of the macrostep flow in combination with local growth speeds, as indicated by the striations shown, for example, in Fig. 7(*a*). An overgrowth of a fast and large macrostep can cause the MPs to bend. Besides mechanisms that can cause a bending of MPs, their mixed character, which includes a screw and edge part, leads to a change of initial MP propagation during growth, which is considered to be constant rather than abrupt.

### 4.2. Dependence of crystal lattice distortion on the dislocation arrangement

RSMs were recorded in the same symmetrical reflection 0004 at selected positions of the wafer areas ‘A’ to ‘D’ on A1 and A2. With the aid of RSMs, one can directly distinguish crystal lattice tilt or strain in the measured area by considering the diffracted intensity distribution along the reciprocal-space coordinates *q*_⊥_ and *q*_||_. The following RSMs always represent an integral diffracted intensity distribution of the whole 5 × 12 mm area shown in the back-reflection topographs. Because of the high density, variety and interactions of dislocations visible in the back-reflection topographs it is not possible to measure the effect of one single dislocation or a specific dislocation type. Furthermore, the effects of dislocation arrangements on the local crystal lattice could be contributed by both the thermal strain and the dislocation. Elimination of the sample’s thermal strain by annealing for a long time was not performed in this study; however, it would be interesting to check the potential correlation for a future research project. The RSM results of the following measurement positions are listed by their similarities of dislocation arrangement and lattice influence.

#### 4.2.1. Local dislocation arrangement causing crystal lattice tilt

**Figure 4:**
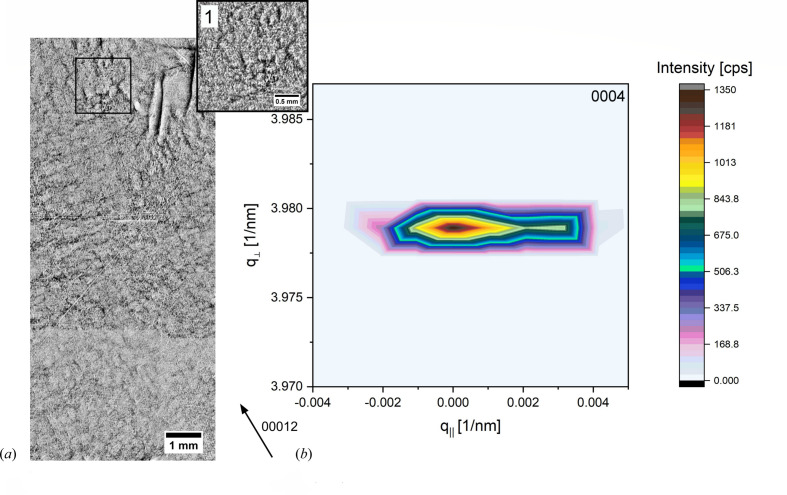
(*a*) A 00012 back-reflection topograph showing the dislocation content of an RSM measurement position on wafer A1, area ‘D’, revealing a regularly arranged BPD network that is homogeneously distributed. A zoomed-in section (1) verifies no appearance of TSDs. (*b*) The corresponding RSM recorded in 0004 reflection shows an expansion of the diffracted intensity distribution along *q*_||_, indicating crystal lattice tilt.

On area ‘D’ of wafer A1, an RSM was recorded in 0004. The 00012 topograph of that position in Fig. 4 ▸(*a*) shows a regular arranged and homogeneously distributed BPD network. Occasionally, there are single TSDs present, in connection to the BPD network, but they are in the minority. Additionally, two white spots can be identified as MPs in connection to short SAGBs. The corresponding RSM in Fig. 4 ▸(*b*) shows a diffracted intensity distribution with an asymmetrical distribution around its maximum. The continuous elongation along the *q*_||_ axis indicates dominating crystal lattice tilt mainly arising from the edge component of the BPD network. There is no diffuse scattering around the diffracted intensity peak visible, indicating a low crystal lattice relaxation. This behaviour changes in areas where besides an edge-component dislocation network (BPD, SAGB) TDs are present in a moderate-to-high density.

#### 4.2.2. Influence of different dislocation arrangements on crystal lattice strain and tilt

**Figure 5:**
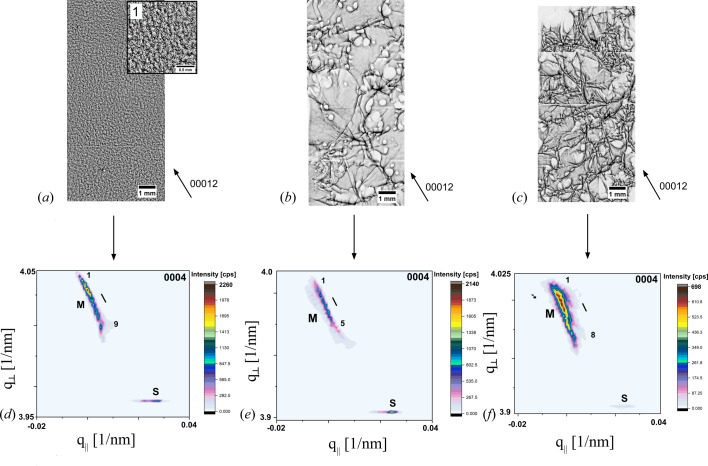
(*a*), (*c*), (*e*) 00012 topographic images of different HRXRD measurement positions with a high variety and density of dislocation arrangements. (*b*), (*d*), (*f*) Corresponding recorded RSMs exhibiting the same principle split into two diffracted intensity distributions, denoted as ‘M’ and ‘S’, despite different dislocation arrangements.

Besides an influence of dislocations causing pure crystal lattice tilt, there are also inherent dislocation arrangements causing lattice tilt and strain. The different positions on A1 and A2 chosen for RSM measurements show different dislocation arrangements in their 00012 topographs in Figs. 5 ▸(*a*), 5 ▸(*b*) and 5 ▸(*c*) but a similar RSM arrangement. The 00012 topograph of an RSM measurement on a wafer area between ‘B’ and ‘D’ on A2 [Fig. 5 ▸(*a*)] shows a regular pattern of a BPD network that is homogeneously distributed. In contrast to the topograph of the previous measurement, there is a connection of the BPD network to a high density of homogeneously distributed TSDs, which act as pinning points for the BPD network. The 00012 topograph of the second measurement position between wafer area ‘B’ and the wafer’s border on A1 [Fig. 5 ▸(*b*)] shows mainly a high number of large-diameter white spots identified as MPs in connection to strongly pronounced SAGBs. These MPs appear to be mostly isolated and show a comparable large white-spot diameter. In the rather uniform grey contrast areas between the strongly pronounced SAGBs and MPs, a BPD network can be assumed to exist, which is not fully depicted due to the dominating SAGB orientation contrast. The 00012 topograph of the third measurement position at the wafer’s border on A2 [Fig. 5 ▸(*c*)] shows a strong increase of the dislocation content and density. Due to the strong overlap of dislocation features, it is not possible to identifiy every individual type. However, an increase in density of SAGBs is certain. The RSMs of all three measurement positions in Figs. 5 ▸(*d*), 5 ▸(*e*) and 5 ▸(*f*) show the same arrangement with small deviations.

All RSMs show a splitting up into two diffracted intensity distributions, where the main one (M) is normalized at *q*_||_ = 0 and the second one (S) is normalized at a different *q*_||_ value. The two are clearly separated by a certain amount of strain and tilt. The main diffracted intensity distribution ‘M’ shows several subordinate diffraction maxima, arranged close to each other in a diagonal arrangement [Fig. 5 ▸(*d*), 1–9)]. Thus, they are also separated by crystal lattice strain and tilt. The second diffracted intensity distribution ‘S’ shows, in all three cases, one maximum surrounded by an asymmetrical intensity distribution and a clear elongation along *q*_||_, which indicates dominating crystal lattice tilt. This appearance fits with the RSM in Fig. 4 ▸(*b*) and thus arises from the contribution of the BPD network, inducing crystal lattice tilt. For all RSMs of the same type, the stress and tilt components between subordinate maxima (labelled by numbers) of ‘M’ and between the two separated diffraction intensity distributions ‘M’ and ‘S’ were calculated according to equations (1) to (4) [Disp-formula fd3] [Disp-formula fd4]. The main diffracted intensity distribution ‘M’ of the RSMs at the first measurement position in Fig. 5 ▸(*d*) shows a symmetrical intensity distribution around each subordinate maximum, pointing to both crystal lattice strain and tilt in the range of 0.44 to 1.66 GPa for stress and −0.39 to 1.44 arcseconds for tilt between subordinate maxima. The two diffracted intensity distributions ‘M’ and ‘S’ are separated by stress and tilt values of 8.85 GPa and 21.45 arcseconds. The second RSM shown in Fig. 5 ▸(*e*) shows again a rather symmetrical intensity distribution around the subordinate maxima 1–5 of ‘M’. Despite the influence of MPs, which have a larger internal strain compared with single TSDs, the effect on the crystal lattice is similar to the previous measurement shown in Fig. 5 ▸(*d*). The stress and tilt values between the subordinate maxima 1–5 lie in the range of 0.13 to 1.3 GPa for stress and −0.41 to 1.69 arcseconds for tilt. Area ‘S’ shows the highest intensity with a clear expansion along *q*_||_ and can be again assigned to a BPD network, which is assumed to be located in the grey areas between MPs and SAGBs shown on the 00012 topograph in Fig. 5 ▸(*b*). The crystal lattice stress and tilt values between the two diffracted intensity distributions ‘M’ and ‘S’ in Fig. 5 ▸(*e*) are 9.23 GPa and 20.2 arcseconds. In contrast to the first two measurement positions discussed so far, the main diffracted intensity distribution ‘M’ of the third RSM in Fig. 5 ▸(*f*) shows a stronger expansion along *q*_||_ around each subordinate maximum (1–8), pointing to an increase in crystal lattice tilt. Since the dislocation density in the measured area is too high to assign all features to the dislocation type, the increase in lattice tilt can only be correlated to the increasing density of SAGBs. Thus, the tilt-value range between the subordinate maxima 1–8 increased and ranges from 0.16 to 1.69 arcseconds. The value range for the stress values between the subordinate maxima of ‘M’ remains in a similar range of 0.35 to 1.29 GPa, which is comparable to that of the first two RSMs in Figs. 5 ▸(*d*) and 5 ▸(*e*). The crystal lattice stress and tilt between the two diffracted intensity distributions ‘M’ and ‘S’ are 8.79 GPa and 19.94 arcseconds and are therefore also in a comparable range to that of the first two RSMs.

Despite a different dislocation arrangement, comparing the values of all three RSM measurements shown in Fig. 5 ▸, it becomes apparent that the crystal lattice stress and tilt between subordinate maxima of the diffracted intensity distribution ‘M’ (denoted by numbers) and between ‘M’ and ‘S’ are all of the same order of magnitude. Moreover, the values between ‘M’ and ‘S’ show less deviation than the values between the subordinate maxima of ‘M’. Most importantly, a high number and density of TSDs in combination with an edge dislocation network has the same effect on the crystal lattice as a comparable region consisting of a low-to-moderate density of MPs, where the terms high, moderate and low refer to the compared number of dislocations (MPs or TSDs) within the measurement area of 65 mm^2^. In the case of the area shown in Fig. 5 ▸(*a*), the number of single TSDs was counted at 2764, whereas in Fig. 5 ▸(*b*) the number of MPs was counted at 103. Some of the white-spot features overlap with others, making it difficult to estimate the exact number of MPs in the given area, but a strong decrease in density compared with the number of TSDs in Fig. 5 ▸(*a*) is certain.

#### 4.2.3. Influence of the dislocation’s orientation on the crystal lattice

**Figure 6:**
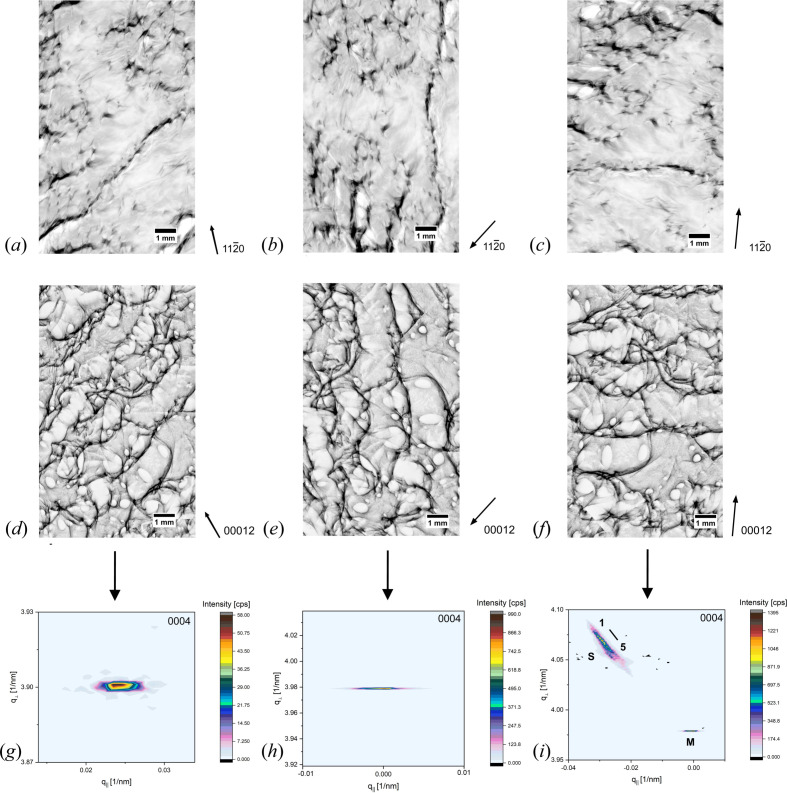
RSM measurement at different rotations about the same sample surface point to align the preferential direction of SAGBs to 45°, parallel or perpendicular to the longitudinal extension of the HRXRD focal beam area. (*a*)–(*c*) 11 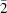 0 transmission topographs showing a pronounced SAGB network. (*d*)–(*f*) Corresponding 00012 topographs at the same sample position and rotations revealing a high density of MPs besides the SAGB network. (*g*)–(*j*) RSMs recorded at different sample rotations demonstrating a change of the diffracted intensity distribution depending on the dislocations’ orientation.

At the border of wafer A1, in the area showing dominating SAGBs aligned along [1 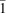 00], several RSMs were recorded in 0004 reflection but at different sample rotations, and thus the dislocation orientation along the scanning direction changes. The sample was rotated in a way that the preferential direction of SAGBs was set to 45°, parallel and perpendicular to the longitudinal extension of the HRXRD focal beam area (5 mm × 12 mm), which is perpendicular to the scanning direction. To confirm the preferential direction of SAGBs, transmission topographs were chosen for comparison. Fig. 6 ▸ shows a comparison of the measurement position in 11 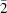 0 transmission [Figs. 6 ▸(*a*)–6 ▸(*c*)], 00012 back-reflection topographs [Figs. 6 ▸(*d*)–6 ▸(*f*)] and the corresponding RSMs recorded in 0004 at the different sample rotations [Figs. 6 ▸(*g*)–6 ▸(*i*)]. The transmission topographs show a more pronounced contrast of SAGBs and the appearance of a large number of pairs of short and parallel dark contrast lines, indicating the positions of MPs. However, contrast features representing a distinct dislocation type in the transmission topographs overlap with other features and appear to be less pronounced than in the back-reflection topographs. Furthermore, the 11 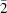 0 transmission topographs in Figs. 6 ▸(*a*)–6 ▸(*c*) reveal no clear appearance of BPDs, but they can be smudged by the high dislocation density and superimposition of different dislocation features with stronger contrast. However, a disappearance or strong decrease of the BPD density is again in good agreement with the decreased shear stress at the border region. The corresponding 00012 back-reflection topographs in Figs. 6 ▸(*d*)–6 ▸(*f*) reveal a high number of large-diameter white spots with an elliptical outline, which can be assigned to MPs. Most of them show a connection to SAGBs, and only a few of them appear to be isolated. There is no visible difference in the SAGB preferential direction between the transmission and back-reflection topographs. Furthermore, both geometries reveal regions directly attached to the SAGBs that show a lack of diffracted intensity, corresponding to orientation-contrast-dependent diffraction loss. Although the measurement position on the wafer remains the same, the RSMs recorded in 0004 differ according to the sample’s rotation. The RSM recorded at 45° in Fig. 6 ▸(*g*) shows one diffracted intensity distribution symmetrically surrounding its maximum. It shows a stronger elongation along *q*_||_ and therefore points to a continuous crystal lattice tilt. Nevertheless, there is still a sufficient expansion in *q*_⊥_ visible, pointing to a certain portion of lattice strain. The second RSM in Fig. 6 ▸(*h*), recorded with the longitudinal extension of the focal area parallel to the SAGB lineation, shows again one diffracted intensity distribution asymmetrically surrounding its maximum but with a clear expansion along *q*_||_, pointing to pure crystal lattice tilt arising from the orientation of SAGBs. In this sample rotation, a maximum of the crystal lattice tilt is achieved by scanning along differently oriented areas separated by SAGBs, and strain is negligible.

The RSM of the last sample rotation in Fig. 6 ▸(*i*), having the SAGB lineation perpendicular to the longitudinal extension of the focal beam area, shows a completely different RSM arrangement. A splitting up into two diffracted intensity distributions, ‘M’ and ‘S’, similar to the RSMs discussed in Figs. 5 ▸(*d*)–5 ▸(*f*), is also shown. The two diffracted intensity distributions, ‘M’ and ‘S’, are separated by a stress and tilt of 9.56 GPa and −23.6 arcseconds, where both values are slightly increased compared with the values of previous RSMs that are of the same type. The main diffracted intensity distribution, ‘M’, shows one maximum surrounded by a continuous asymmetrical intensity distribution. There is a significant elongation along *q*_||_ visible, pointing to dominating crystal lattice tilt. However, the extension of the diffracted intensity distribution shrank compared with the RSMs of the first two rotations on wafer A1 in Figs. 6 ▸(*g*) and 6 ▸(*h*). The second diffracted intensity distribution, ‘S’, shows again several subordinate maxima, which are separated by a certain amount of crystal lattice stress and tilt in the range of 0.19 to 0.26 GPa and −4.06 to 11.26 arcseconds. This is a comparable stress range to the subordinate maxima of the RSM regions shown in Figs. 5 ▸(*d*), 5 ▸(*e*) and 5 ▸(*f*), but the tilt range is clearly increased, which is in accordance with the high density of SAGBs. Despite the same wafer position and dislocation arrangement applying to all three measurements at different rotations, the recorded RSM changes according to the dislocation’s orientation. Taking into account that, due to the sample rotation, a slightly different dislocation arrangement within the focal beam area contributes to the signal, the change of the RSM arrangement depends on the scanning direction along different dislocations. Thus, dislocations have a different effect on the crystal lattice at different crystallographic directions. Perpendicular to the SAGBs’ preferential direction, a maximum of crystal lattice tilt is measured, whereas an intermediate position (45°) leads to crystal lattice strain and tilt. A splitting up into two diffraction areas, ‘M’ and ‘S’, is in accordance along the SAGBs’ preferential direction and, moreover, a specific MP orientation. Despite the same arrangement of diffracted intensity distributions for RSMs that show a splitting up into areas ‘M’ and ‘S’ and a comparable stress/tilt range, the values between subordinate maxima slightly differ for those with an increased SAGB density. All diffracted intensity distributions with an expansion along *q*_||_ indicating lattice tilt appear to be continuous, pointing to high-mosaicity areas in which small neighbouring sections with slightly different orientations are present instead of large areas that are clearly separated by lattice tilt.

#### 4.2.4. A proposed model for TDs causing strain and tilt

**Figure 7:**
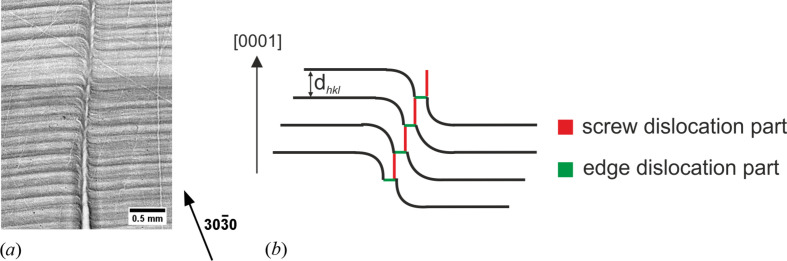
(*a*) A 303 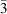 0 back-reflection topograph along a mixed-type MP. Growth striations indicate the deformation, with the resulting tilt and strain in the surrounding lattice around the MP’s core. (*b*) A model of a single mixed-type MP separated into screw and edge components, which leads to a separation into subordinate diffracted intensity maxima in an RSM, corresponding to the MP’s edge and screw dislocation parts. The path of the screw dislocation part is parallel to the main growth direction in [0001] and the edge dislocation part propagates in the (0001) basal plane. X-ray diffraction is also possible close to the MP core at the same angle as for undisturbed lattice planes, and the difference between the edge and screw dislocation parts can be quantified.

Comparing the RSM results recorded at different sample rotations, an influence of the dislocation’s orientation on the crystal lattice is certain. As shown in Figs. 5 ▸ and 6 ▸, for a specific dislocation arrangement but also for a specific dislocation orientation there is a separation into two diffraction areas, ‘M’ and ‘S’, which are separated by strain and tilt and which can further be quantified. This separation takes place in areas with a high dislocation density and a dislocation arrangement in which TDs are in combination with an edge dislocation network. In Fig. 4 ▸ it was shown that the occurrence of a BPD network, despite the fact that it can be non-pure, shows in the RSM a continuous lattice tilt. Thus, the subordinate maxima of one peak are presumably caused by a bunch of single TDs that are of a mixed dislocation type and are clearly separated in screw and edge components, where the screw part is propagating along the [0001] growth direction and the edge part is in the (0001) basal plane. Fig. 7 ▸(*a*) displays a 30 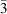 0 back-reflection topograph at 7.29 keV with a Bragg angle of 78° from a longitudinal wafer cut (along the growth direction), showing the dislocation path of a mixed-type MP propagating nearly along the growth direction. Growth striations indicate the deformation, with the resulting tilt and strain in the surrounding lattice around the MP’s core. Considering the arrangement of the displacement field around an MP core, as shown in Fig. 7 ▸(*b*), a distinction between the screw and edge dislocation parts is possible for MPs showing a clear separation in the screw and edge dislocation parts. For these MPs, the displaced lattice planes around the core between each screw-to-edge step are clearly pronounced and at a suitable angle to diffract X-rays, which also diffract at undisturbed lattice planes. Therefore, X-ray diffraction is possible close to the MP core at the same angle as for undisturbed lattice planes, and the difference between the edge and screw dislocation parts can be quantified. For a sufficiently large set of these MPs that have a suitable angle and direction, the tilt and strain differences around the MP’s core between the edge and screw dislocation parts result in a peak splitting of the corresponding RSM, and the amount of strain and tilt can be quantified. As already discussed, a sufficiently large density of TSDs, which fulfils the same prerequisites as the low-to-moderate density of MPs, has the same measurable effect on the crystal lattice [as shown in Figs. 5 ▸(*a*) and 5 ▸(*d*)], and, moreover, the strain and tilt difference between the edge and screw dislocation parts are of the same order of magnitude.

## 5. Conclusions

On the basis of full wafer mappings in back-reflection geometry, it was possible to obtain an overview of the dislocation arrangement concerning types, density and homogeneous distribution, and, moreover, a possible change of the dislocation arrangement could be followed in the growth direction. Therefore, wafer areas consisting of a similar dislocation arrangement could be identified. Despite a distance of 9.4 mm between wafers A1 and A2 of the same crystal, these areas remain unchanged along the growth direction. Nevertheless, there are local changes visible regarding the position of single TSDs or MPs between the wafers, pointing to a change of their dislocation path. Having a similar resolution to conventional photographic film topography, the imaging of large wafers with a CCD camera system looks promising, since photographic films are becoming outdated and could be unavailable in the future. For the first time, a systematic analysis of different dislocation arrangements causing crystal lattice strain or tilt was performed. The results of the RSM measurements confirm a local change that depends not only on the dislocation types and density but also on their orientation. There is always a splitting up into two diffracted intensity distributions for measurement positions containing a sufficient number of TDs in connection with an edge-component dislocation network, namely BPDs or SAGBs. Most importantly, a high density of TSDs connected with a network has the same measurable effect on the crystal lattice as a low-to-moderate density of MPs connected with the same network. Therefore, if the framework of dislocations surrounding TSDs is similar, the effects on the crystal lattice can be directly transferred from MPs to TSDs. Furthermore, the strain and tilt differences between the edge and screw dislocation parts lie in the same order of magnitude. A possible model was also introduced to show how the TDs’ orientation and inclination can cause a subdivision of one diffracted intensity distribution into several subordinate maxima.
